# Supplementary material for: The Fungal Endophyte Penicillium olsonii ML37 Reduces Fusarium Head Blight by Local Induced Resistance in Wheat Spikes
Source: J Fungi (Basel). 2022 Mar 25;8(4):345. doi: 10.3390/jof8040345 (PMC9025337; doi:10.3390/jof8040345)
Supplement: Supplementary file 1 [file jof-08-00345-s001.zip › Rojas_et_al-SI_JoF.pdf]

Journal of
Fungi

MDPI

Journal of Fungi **Supporting Information**
Article title: The fungal endophyte *Penicillium olsonii* ML37 reduces Fusarium head blight by local induced resistance in wheat spikes
Authors: Edward C. Rojas, Birgit Jensen, Hans J. L. Jørgensen, Meike Latz, Pilar Esteban & David B. Collinge
Article acceptance date: Click here to enter a date.

The following Supporting Information is available for this article:

Figure S1 Transcriptome changes in wheat spikes at 48 h after *P. olsonii* ML37 inoculation. Expression of five defence related responses: *PR*-genes and several genes reported to be highly active during anthesis in wheat. Boxplot shows range and median of mean tpm for each gene. Statistically significant differences (q<0.01 using Walt test) are denoted as '**'. Deep green colour denotes genes differentially expressed. Light green colour denotes non-differentially expressed genes

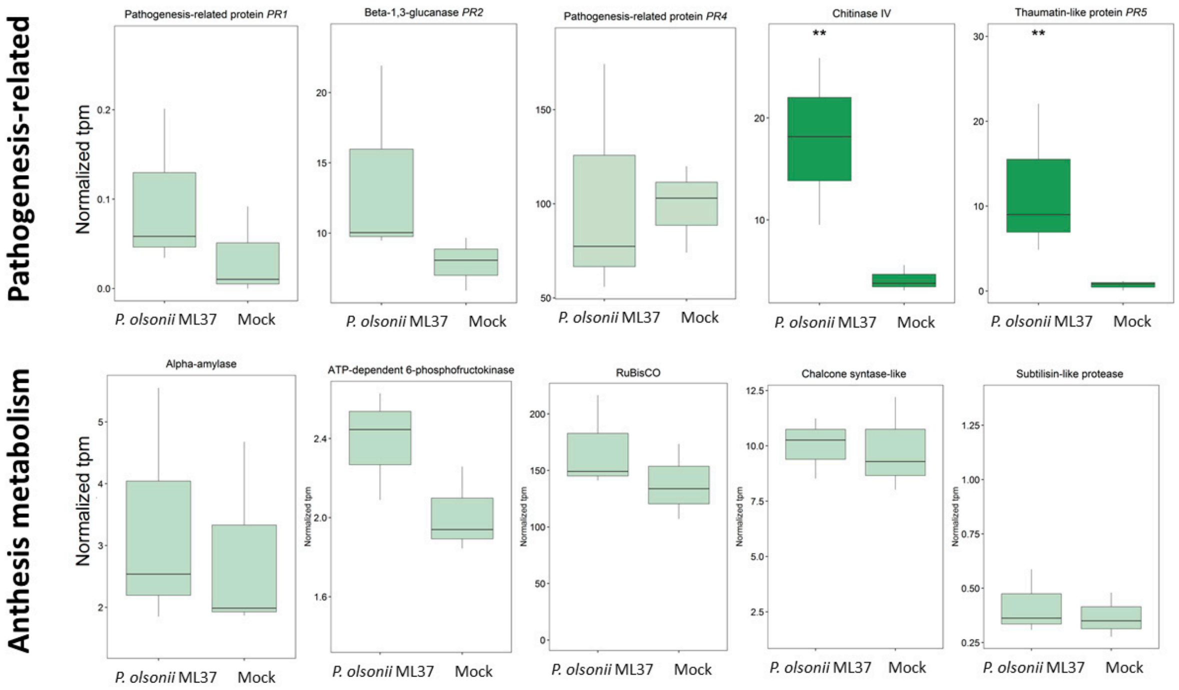

Figure S2 A: Specific β-1,3-glucanase activity in wheat spikes treated with *P. olsonii* ML37 or water at 24, 48 and 72 hpi. **B.** β-1,3-glucanase activity in ML37-treated spikes at 24, 48 and 72 h after *Fusarium* inoculation. Statistically significant differences (P<0.05 using a generalized linear model) are denoted as '*'.

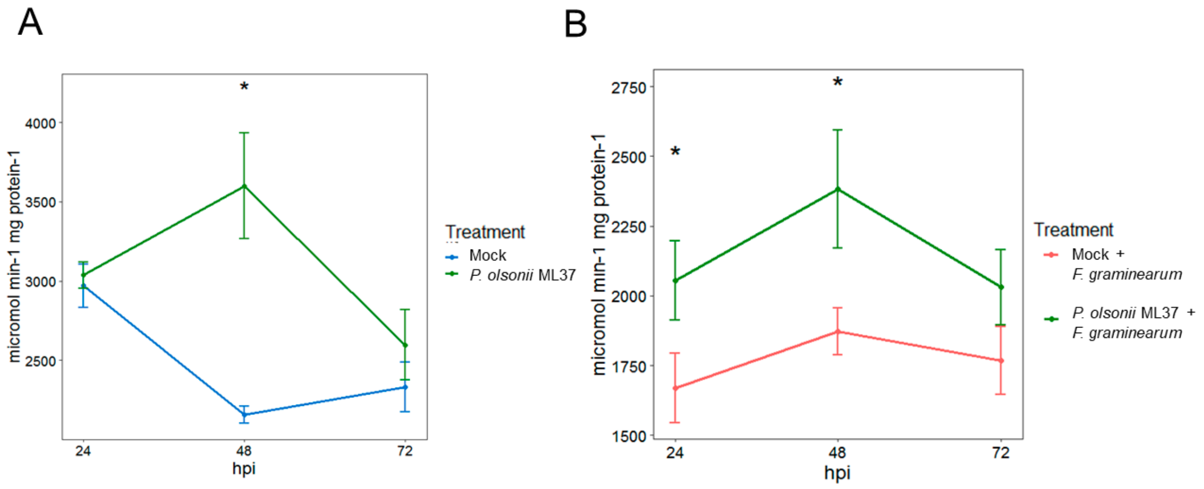

Figure S3 Expression of five different chitinase genes and five genes involved in specialised metabolite production in wheat in *P. olsonii* ML37-treated plants at 24 h after *Fusarium* inoculation. Boxplot shows range and median of mean tpm for each gene. Statistically significant differences (q<0.01 using Walt test) are denoted as '**'. Deep green colour denotes genes differentially expressed. Light green colour denotes non-differentially expressed genes.

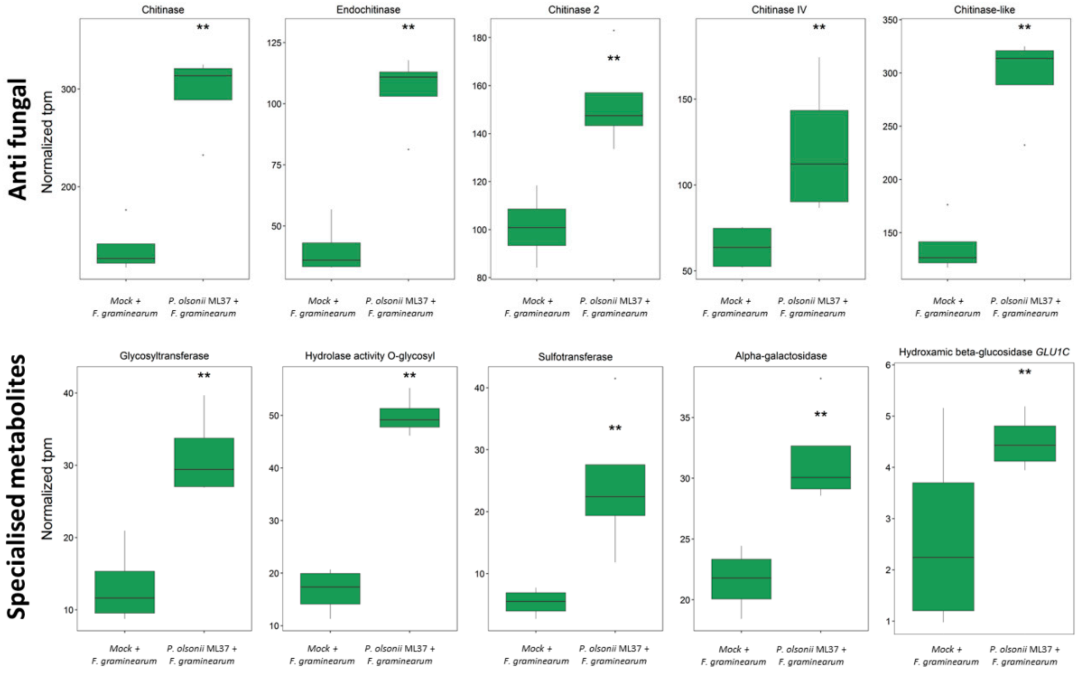

Journal of Fungi

MDPI

Figure S4 Transcriptome changes in *Fusarium graminearum* during *P. olsonii* ML37-mediated biocontrol of FHB at 24 and 72 hpi. **A (left).** Normalised transcripts per million (tpm) of a cluster of genes with negative regulation in ML37-treated plants at 3 dpi hpi. Grey lines represent mean expression of transcripts, Blue line represents total cluster mean. **A (right).** Abundant gene ontology terms within each cluster (none of them were significantly enriched). Bar plots represent –log(*P*-value) of each GO term. **B (left).** Normalised tpm of a cluster of genes negatively regulated in ML37-treated plants at both time points. **B (right)**. Abundant GO terms. Gene ontology conventions: BP: Biological process, CC: Cell component and MF: Molecular function. **C.** Expression of three genes involved in stress responses. Boxplot shows range and median of normalized tpm for each gene. Statistically significant differences (q<0.01 using Walt test) are denoted as '**'

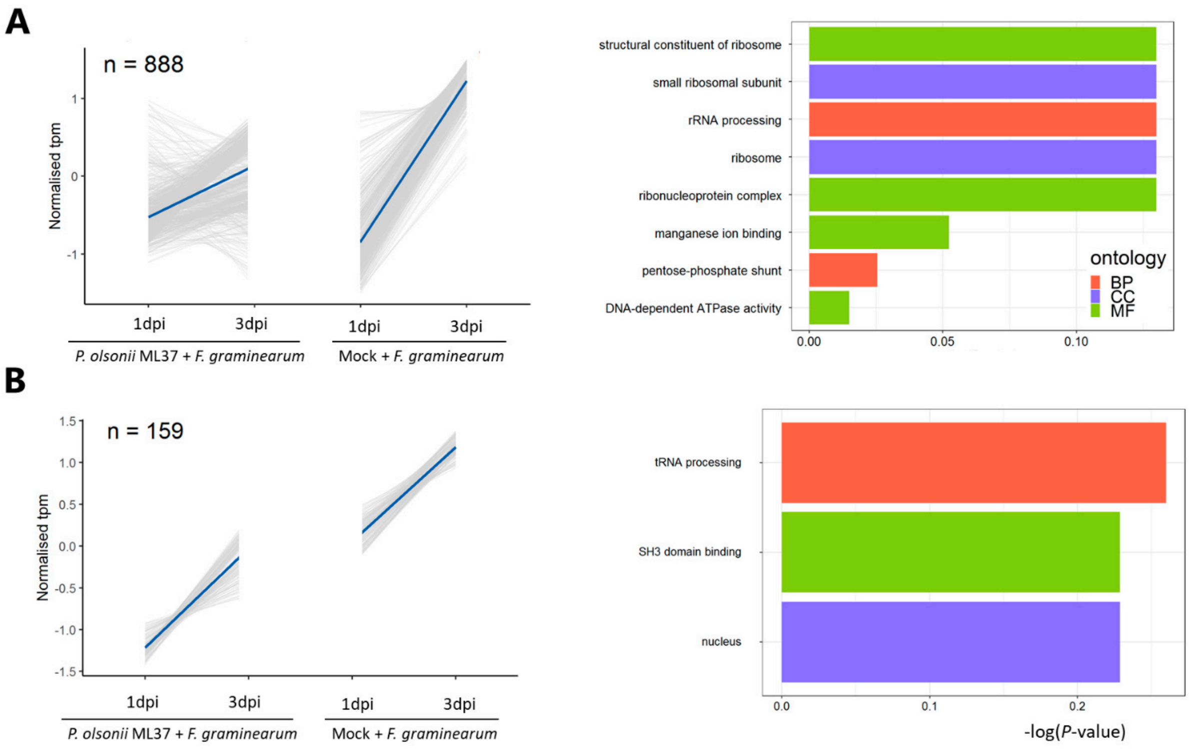

Journal of Fungi

MDPI

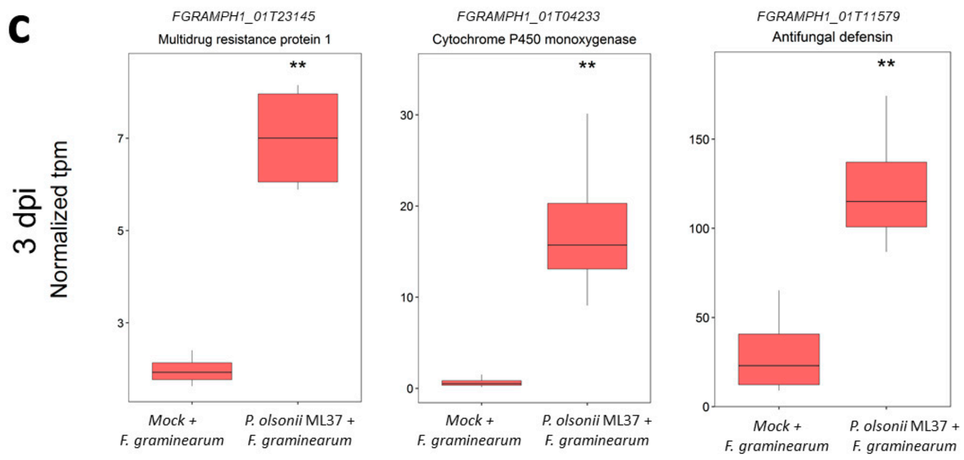

Table S1 Differentially expressed genes in wheat at 48h after P. olsonii ML37 inoculation.

Table S2 Differentially expressed genes in wheat - *P. olsonii* ML37 mediated biocontrol of FHB (24hpi)

Table S3 Differentially expressed genes in wheat - *P. olsonii* ML37 mediated biocontrol of FHB (72hpi)

Table S4 Differentially expressed genes in *F. graminearum* during *P. olsonii* ML37-mediated biocontrol of FHB (24hpi)

Table S5 Differentially expressed genes in *F. graminearum* during *P. olsonii* ML37-mediated biocontrol of FHB (72hpi)
